# Irregular Menses

**Published:** 1887-12

**Authors:** Ad. Lippe


### IRREGULAR MENSES.

Menses in morning only:* Sepia.
— — — more profuse: Bovista.
— ceasing in afternoon: Mag-carb.

---

* Under Bovista, Hering gives: "Menses only at night or only in morning." Also, "Menses profuse in morning, scanty during day and night."

Under Carbo an., Jahr gives: "Menses not copious, but last longer than usual, and only flow in the morning," which is similar to Sepia.—Eds.

Menses increasing in afternoon : Puls., Sulph.
— in evening only : Coff., Phell.
— during day only : Caust.
— only when walking, not during rest : Lil.-tig.
— only when lying, ceasing when walking : Mag-carb.
— cease when lying : Cact., Caust., Lil.-tig.
— at night only : Bov., Brom.
— — — increased : Amm.-c., Mag.-c., Zinc.

AD. LIPPE.

